# Supplementary material for: The Cytomegalovirus Tegument Protein UL35 Antagonizes Pattern Recognition Receptor-Mediated Type I IFN Transcription
Source: Microorganisms. 2020 May 26;8(6):790. doi: 10.3390/microorganisms8060790 (PMC7356634; doi:10.3390/microorganisms8060790)
Supplement: Supplementary file 1 [file microorganisms-08-00790-s001.zip › Supplementary figures.docx]

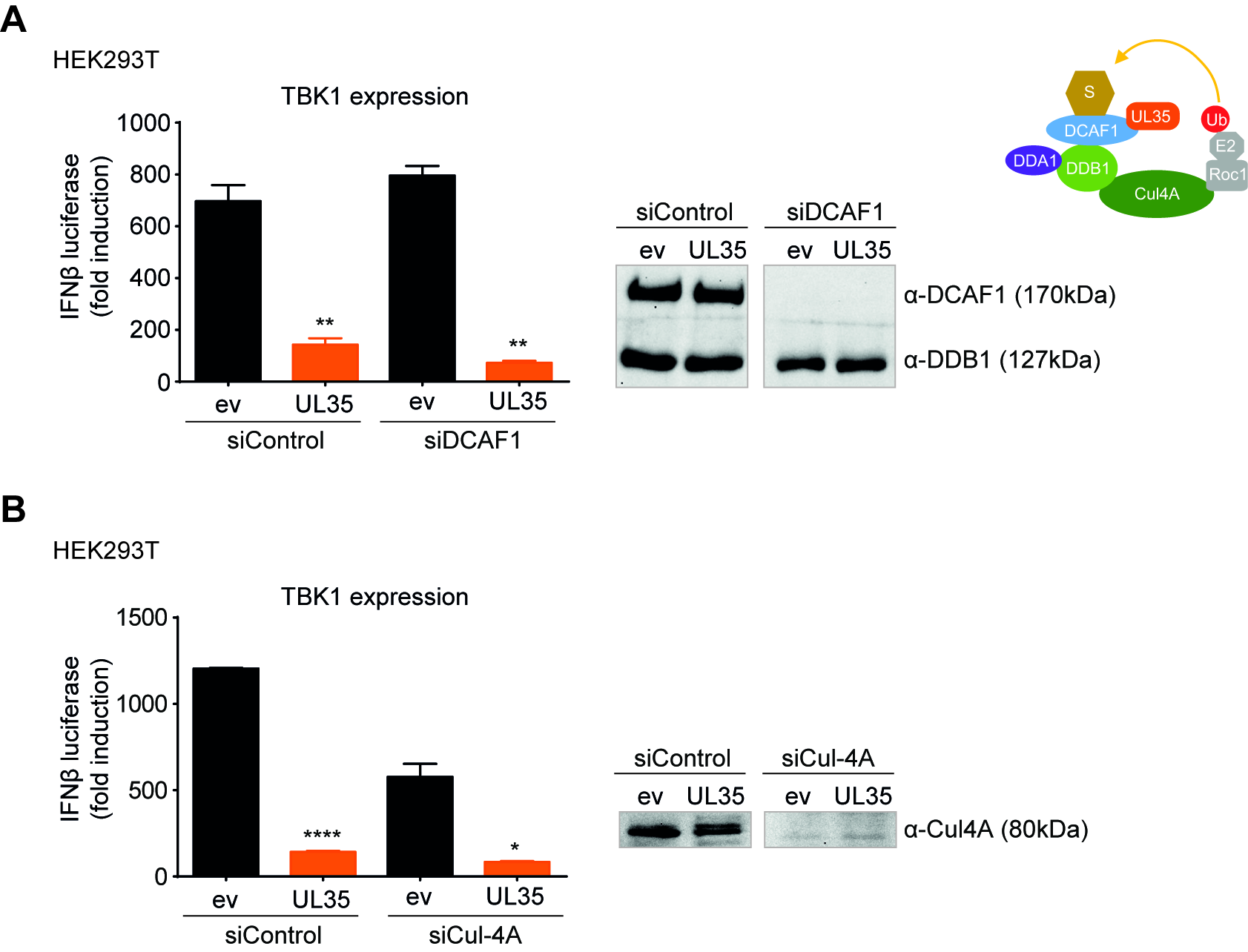


**Figure S1. UL35 inhibits IFNβ transcription independent of DCAF1 or Cul4A.** HEK293T cells were reverse transfected with either control siRNA or siRNA specific for DCAF1 (**A**) or Cul4A (**B**). 48 hours post transfection, cells were co-transfected with IFNβ-Luc, pRL-TK, TBK1 (stimulated) or ev (unstimulated control) and either ev or UL35-HA. 20 hours later, cells were lysed and luciferase activity was measured. Simultaneously, luciferase whole cell lysates were immunoblotted for DCAF1 and DDB1 (**A**) or Cul4A (**B**). (**A-B**) Shown are mean data of two independent experiments ± SD. Student’s t-test (unpaired, two-tailed), UL35 was compared to ev. *p<0.05, **p<0.01, ****p<0.0001.


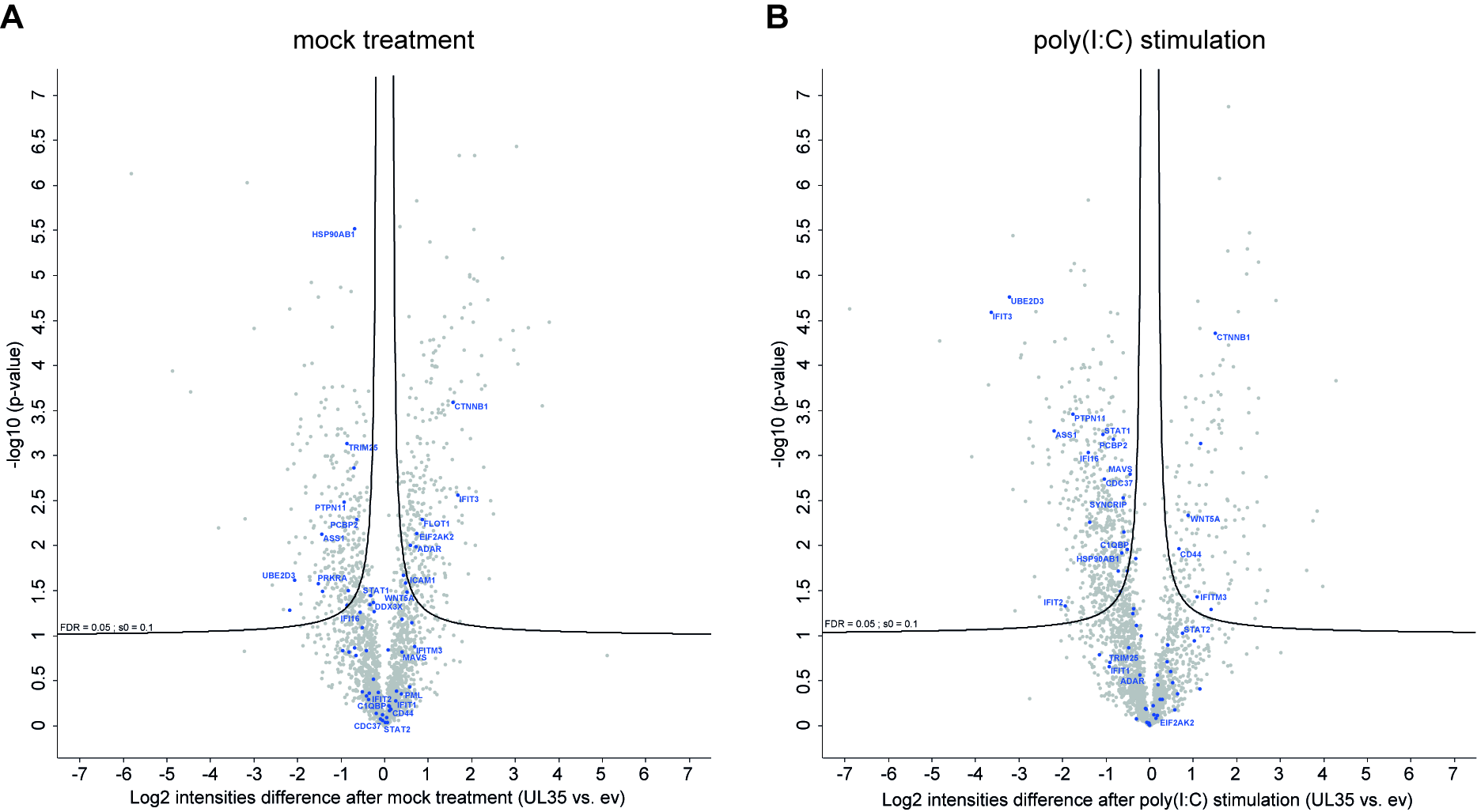


**Figure S2. Whole cell proteomics of HFF-1 stably expressing ev or UL35.** HFF-1 stably expressing ev or UL35 were stimulated by transfection of poly(I:C) or medium only for 4 hours. Whole cell lysates were prepared and peptides were subjected to LC-MS/MS for proteomic analysis. Proteome changes between ev- and UL35-expressing cells after mock treatment (**A**) and poly(I:C) stimulation (**B**) are shown in the volcano plot. Combined data of four independent experiments are shown. Student’s *t*-test (unpaired, two tailed) was applied to compare UL35 to ev.
